# The Hospitals, &c., of New York

**Published:** 1860-01

**Authors:** 


### EDITORIAL DEPARTMENT.

### *The Hospitals, etc., of New York.*

New York Hospital.—The buildings of this institution, situated on the most prominent thoroughfare of our great city, have for many years presented themselves to the gaze of passers-by; the ivy-grown stone walls, as seen from Broadway, shaded by time-honored elms, appearing suggestive of retirement and quiet amidst the din of busy life around.

The charter of this hospital was granted by George III., in the year 1771; and it was incorporated under the name of the "Society of the Hospital in the City of New York, in America." The Mayor, Recorder, and Aldermen of the city, the Rector of Trinity Church, several clergymen, the President of King's (now Columbia) College, and a number of the most respectable inhabitants of the city, were named as members. Twenty-six governors were appointed for the management of the affairs and business of the institution, who held their first meeting on the 25th of July, 1771. The first building erected was destroyed by fire when nearly completed, and the effects of the war on the condition of the city, and the general derangement of affairs, prevented any attention to the institution for some years, and it was not until the beginning of 1791 that the house was in a proper condition to receive patients,—when eighteen were admitted.

In 1806, a large and substantial building was erected on the southern part of the hospital ground, for the purpose of an asylum for insane patients. It was rendered useless for that object in 1821, by the establishment of the large institution at Bloomingdale. In 1825, it was thoroughly repaired and remodeled as a Hospital for Seamen. Another edifice was completed in 1841, being placed on the north side of the grounds, and corresponding in general appearance with the marine building. The large center building, though originally constructed on the best plan, was deficient in some of the minor arrangements conducive to the health of the patients. These defects were gradually remedied by various alterations and improvements. In 1843, the Croton water was introduced into the several buildings, thereby adding much to the comfort and health of the inmates.

An improved plan for heating the buildings by steam, and a thorough arrangement for ventilation, were likewise introduced.

The marine building being inadequate to the growing wants of the institution, it was demolished, and a new structure erected, which was completed in 1855. It is known as the "South Building;" and as a complete and commodious hospital edifice, it can perhaps be nowhere surpassed. Its cost was $140,000.

The library, which was founded in 1796, now consists of more than 6,000 volumes, confined entirely to medicine and surgery, and those collateral branches of science especially connected with the healing art. It is believed to be as useful and complete as such a library can be made with that number of volumes, and contains many of the most valuable works on anatomy and natural history.

About 1840, the commencement of a pathological cabinet was made. It was formed from the more remarkable cases of morbid anatomy occurring in the practice of the hospital; and it has increased rapidly in extent and value for purposes of science and instruction. A roomy stone outbuilding was fitted up to contain the pathological cabinet, where it is now arranged to great advantage.

Clinical teaching, both surgical and medical, has long been regularly given, on the cases arising in the practice of the house, to the medical students admitted to attend. This has been found here, as in other cities, the most valuable adjunct to the public instruction of teachers in the medical schools; and the officers of the hospital have been long zealous to make this branch of instruction commensurate with the advance of medical knowledge and the increased demand for higher professional education. The present physicians and surgeons, as well as their immediate predecessors, have spared no pains to render the institution under their charge effective to this end, by furnishing students with every facility for acquiring a practical knowledge of the nature and treatment of disease. The price of yearly tickets of admission for students was formerly five dollars; but, of late, tickets are issued gratuitously to gentlemen who show proof that they are regular students of medicine. Clinical lectures are given in the wards by the attending surgeons and physicians, six days in the week, at half-past one P. M. Operations that can be delayed are always performed in the theatre, before the students.

This hospital has never been used to supply in any degree the place of a poor-house or infirmary for the merely destitute. It was from the first devoted to the reception and care of patients whose cases, in the judgment of the attending physicians, admitted of a reasonable expectation of cure, or of some substantial relief. The only exception to this rule, but which has of late years become a frequent one, is that persons receiving any sudden injuries, such as constantly occur in a populous city, are always received, whatever may be their condition. There are three several classes of persons received:—First, those without means of payment, who are taken in according to the judgment of a committee on their several cases. These patients, supported gratuitously, constitute an average of about 40 per cent. of the whole number under treatment. Second, *seamen*, paid for from the hospital money paid under the laws of the United States. The experience of many years has proved that a larger number of this valuable class of citizens can be thus received with a greater degree of comfort, and with superior attendance and medical and surgical aid of a higher class, than the same aggregate expense could obtain for them if applied in any other way. Third, pay-patients; these at a rate scarcely sufficient to repay the mere expense of support, so that thus the best medical and surgical skill, equal to any which the wealthiest citizen can command, is placed within the reach of many, who, though not in the situation of paupers, would find their limited means soon exhausted by paid professional services. Mutual benefit clubs, benevolent societies, and similar associations, continually avail themselves largely of these advantages.

In both of these last-named classes, the benefits of this hospital have been largely diffused far beyond the bounds of the city, or even of the State. Seamen from all parts of the Union, having paid hospital money at any port, have always filled the wards of the Marine Department; while patients suffering under diseases requiring peculiar skill or care (especially those of a surgical nature), such as they could not find near them, continually resort to this establishment from distant parts.

From the 1st of February, 1792, shortly after the re-opening of the hospital, to January 1st, 1856, 106,111 patients have been received, of whom 77,390 have been discharged *cured*, and 4,768 as *relieved*. Out of the 106,111 received, 10,893 have died, among which are included a considerable number in each year,—and in late years, above 150 every year,—brought in in a dying state, from casualties in the city. Thus, 82,058 have been cured or relieved out of 106,111 who received the benefits of the institution. But in later years, since the improved hospital arrangements as to water, warmth, air, &c., have added their efficient aid to medical skill, the proportion of deaths is much less than that indicated by the above aggregates. Deducting cases of casualties, when persons brought in were in a hopeless state, and died within a few hours, the ratio of deaths in 1853 was $5\frac{57}{100}$, in 1854, $5\frac{85}{100}$, in 1855, $5\frac{79}{100}$ per cent. The number of patients admitted rose gradually with the growth of the city from 566 in 1791, to 1,037 in 1821; then it annually augmented to 1,870 in 1831, and to 2,000 in 1841. During the next ten years the progress was still more rapid, until it reached 3,715 in 1851. During the next five years ending January 1st, 1856, there were 16,948 admissions; or (including the patients that remained at the end of each year) an annual average of about 3,880 patients who received the benefits of the New York Hospital in those years.

Of the several classes of patients above enumerated, during the last five years, about one fourth, or from 22 to 27 per cent. annually, have been persons paid for by themselves or their friends. More than one third, or from 33 to 40 per cent., have been supported gratuitously. The remainder have been seamen, received under the arrangement with the United States Treasury Department.

The position of the hospital, in the center of active business, in the vicinity of the shipping, and near the termination of railroads, has always rendered it the most convenient and great point of resort for relief for surgical cases, as well for strangers as residents. Many of these are sufferers from sudden accidents—from fires, wounds, falls, injuries on board river or ocean vessels in port, or on railroads. In consequence of this location and the increased business throng of the city, the surgical cases in this hospital have regularly and gradually increased both in their numbers and in their proportion to those from other diseases.

Thus, the New York Hospital has become the most extensive school of surgery in this country. The comparative statistics of the medical and surgical departments for the several past years, present a striking evidence of the remark just made as to these classes of patients. In 1854, the proportion of surgical cases to medical was 19¾ to 17, which doubled the average of earlier years. But of the cases under hospital treatment in 1855, there were 1,789 surgical to 1,224 medical, or nearly one third more surgical. Of the surgical cases, 831 arose from sudden casualties; three fourths of them occurring in the part of the city below Broome Street. Many others were from accidents on the railroads terminating in the city.

The report of last year shows the following statistics. The whole number of patients who received the benefits of the hospital as medical or surgical patients during the year 1858, was 2,451.

The number of patients in the several buildings of the hospital in the the city, on the 31st day of December, 1857, was two hundred and twenty-nine, and there were admitted during the year 1858, two thousand two hundred and twenty-two.

| | |
|---|---|
| Of this number there have been cured, - - | 1,506 |
| Relieved, - - - - - - | 294 |
| Discharged at their own request, - - - | 92 |
| " as improper objects, - - | 25 |
| Eloped, or discharged as disorderly, - - | 26 |
| Died, - - - - - - | 274 |
| | 2,217 |
| Remaining 31st December, 1858, - - | 234 |
| Total treated, - - | 2,451 |

Of the whole number of cases under actual treatment during the year 1858, a little less than $5\frac{47}{100}$ per cent. *died*; or, taking the ratio of mortality upon all discharged (as is done in some statistics of this nature), a little above $6\frac{14}{100}$ per cent. died. The amount of cures out of the number discharged, including deaths, shows a proportion of $67\frac{98}{100}$ per cent. discharged *cured*, exclusive of about $13\frac{35}{100}$ per cent. discharged as relieved for the time.

Of the cases under hospital treatment in 1858, 1,502 were surgical, and 949 medical; being about the proportion of $15\frac{8}{10}$ to 10, or over one half more surgical than medical cases. In 1857, the surgical were to the medical as 18 to 10.

Of the 274 deaths, 140 were coroners' cases.

The following is a list of the surgeons and physicians connected with the institution:

*Consulting Surgeons.*—Valentine Mott, M. D.; Richard K. Hoffman, M. D.; Alex. H. Stevens, M. D; Alfred C. Post, M. D.; John C. Cheeseman, M. D.

*Attending Surgeons.*—Gurdon Buck, M. D.; John Watson, M. D.; Thaddeus M. Halsted, M. D.; Thomas M. Markoe, M. D.; Wm. H. Van Buren, M. D.; Willard Parker, M. D.

*Resident Surgeons.*—Joseph J. Hull, M. D.; Henry N. Fisher, M. D.

*Attending Physicians.*—Joseph M. Smith, M. D.; John H. Griscom, M. D.; Henry D. Bulkley, M. D.; Thomas F. Cock, M. D.

*Resident Physician.*—Clarence Cameron, M. D.

The Attending Surgeons and Physicians are appointed by the Board of Governors, and their term of service continues indefinitely. The Residents are appointed also by the Board of Governors. The term of service for each member of the house staff is two years, which is divided into three periods of eight months each. The first period is served as Junior Assistant; the second as Senior Assistant; and the third as Resident. Each

Attending Surgeon and Physician has the privilege of recommending three candidates for the position of Junior Assistant. The candidates are thoroughly examined by the Attending Surgeons and Physicians, as to their medical and general knowledge; and the three who pass the best examination are recommended to the Board of Governors, who appoint them. There being two surgical and one medical division, two Junior Assistants are appointed on the surgical, and one on the medical, every eight months. The Resident Surgeons and Physician are the only medical officers who live in the house.

The Bloomingdale Asylum for the Insane.—As there existed no institution in the State for the reception and cure of the insane, the Governors, at an early period, appropriated apartments in the hospital; but as they were inadequate to the purposes designed, the Asylum at Bloomingdale was opened in 1821.

It is situated on the Bloomingdale Road, and bounded on the northern side by 110th Street. The grounds comprise eighty acres, beautifully laid out, and under a high state of cultivation. The buildings are handsome, spacious, and arranged on the most approved plan.

This institution, when first opened, was a pioneer establishment in this country for the amelioration of the condition of the insane, and the introduction of that improved system of medical and moral treatment which has since become general. As the Asylum had no fund originally for its ordinary support, it had never the means of receiving many patients gratuitously. It did, however, receive insane poor, paid for by towns and counties, for many years, until the erection of the Blackwell's Island Asylum by the Corporation of New York, and of the Utica State Asylum, provided directly for most of such patients. The insane poor then received were provided for at the lowest rate which could support them; but for some years none of that class have been placed here. But a great value of this establishment consists in bringing its benefits within the reach of persons of moderate circumstances; so that the committee intrusted with its management have been enabled to reduce the charge for support and treatment down to a rate much below that which private enterprise could afford, and merely sufficient to defray the expenses of personal support. Thus it has brought the means of probable cure or relief, and always of a comfortable retreat, within the reach of persons of limited circumstances, who can support a child or parent afflicted with mental disease, with better advantage and at less cost than that of the support and care of such persons at home.

The health of the patients, independently of their peculiar malady, has been generally good, and they have been free from any epidemic or local disease. The following table shows the relative state of the Asylum, and result of its practice, for the last ten years.

| YEAR. | ADMITTED. | WHOLE NO. DURING YEAR. | WHOLE NO. DISCHARGED AND DIED. | RECOVERED. | IMPROVED. | NOT IMPROVED. | DIED. | REMAINING AT END OF YEAR. | AVERAGE NUMBER. |
|---|---|---|---|---|---|---|---|---|---|
| 1849 | 95 | 214 | 111 | 44 | 33 | 13 | 21 | 103 | 112 |
| 1850 | 97 | 200 | 90 | 40 | 15 | 7 | 18 | 110 | 110 |
| 1851 | 95 | 205 | 83 | 43 | 20 | 9 | 11 | 122 | 114 |
| 1852 | 104 | 226 | 107 | 47 | 25 | 15 | 18 | 119 | 117 |
| 1853 | 135 | 254 | 130 | 49 | 27 | 32 | 22 | 124 | 133 |
| 1854 | 122 | 246 | 119 | 48 | 29 | 16 | 26 | 127 | 126 |
| 1855 | 107 | 234 | 107 | 52 | 13 | 23 | 19 | 127 | 127 |
| 1856 | 134 | 261 | 117 | 54 | 27 | 17 | 19 | 144 | 140 |
| 1857 | 128 | 272 | 126 | 49 | 37 | 25 | 15 | 146 | 141 |
| 1358 | 112 | 258 | 113 | 34 | 34 | 34 | 11 | 145 | 146 |

The patients are under the care of D. Tilden Brown, M. D., assisted by Charles Corey, jr., M. D., who resides on the premises.

